# SLCV–a supervised learning—computer vision combined strategy for automated muscle fibre detection in cross-sectional images

**DOI:** 10.7717/peerj.7053

**Published:** 2019-07-22

**Authors:** Anika Rettig, Tobias Haase, Alexandr Pletnyov, Benjamin Kohl, Wolfgang Ertel, Max von Kleist, Vikram Sunkara

**Affiliations:** 1 Systems Pharmacology and Disease Control, Freie Universität Berlin, Berlin, Germany; 2 Department of Traumatology and Reconstructive Surgery, Campus Benjamin Franklin, Charité–Universitätsmedizin Berlin, corporate member of Freie Universität Berlin, Humboldt-Universität zu Berlin, and Berlin Institute of Health, Berlin, Germany; 3 Computational Medicine, Zuse Institute Berlin, Berlin, Germany

**Keywords:** Supervised learning, Muscle fibre cross-sectional area, Computer vision, Automated muscle fibre detection

## Abstract

Muscle fibre cross-sectional area (CSA) is an important biomedical measure used to determine the structural composition of skeletal muscle, and it is relevant for tackling research questions in many different fields of research. To date, time consuming and tedious manual delineation of muscle fibres is often used to determine the CSA. Few methods are able to automatically detect muscle fibres in muscle fibre cross-sections to quantify CSA due to challenges posed by variation of brightness and noise in the staining images. In this paper, we introduce the supervised learning-computer vision combined pipeline (SLCV), a robust semi-automatic pipeline for muscle fibre detection, which combines supervised learning (SL) with computer vision (CV). SLCV is adaptable to different staining methods and is quickly and intuitively tunable by the user. We are the first to perform an error analysis with respect to cell count and area, based on which we compare SLCV to the best purely CV-based pipeline in order to identify the contribution of SL and CV steps to muscle fibre detection. Our results obtained on 27 fluorescence-stained cross-sectional images of varying staining quality suggest that combining SL and CV performs significantly better than both SL-based and CV-based methods with regards to both the cell separation- and the area reconstruction error. Furthermore, applying SLCV to our test set images yielded fibre detection results of very high quality, with average sensitivity values of 0.93 or higher on different cluster sizes and an average Dice similarity coefficient of 0.9778.

## Introduction

**Figure 1:**
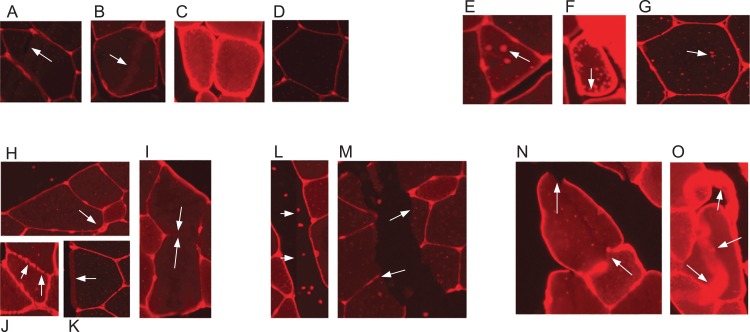
Example sections of WGA fluorescence-stained images. (A–D) Examples for variation in cell tissue intensity. (A) Arrows point at holes in cell tissue which disturb the cell detection process. (B) Intensity variation within one cell. (E–G) Different intensities and distributions of noise. (H–K) Examples for varying border staining intensities. (I) Arrows show irregularities, such as a border between two cells, which is so weakly stained, that it is hardly visible. (L and M) Examples of noise in gap regions. (L) Sudden intensity changes inside the cross section along a vertical line through the image, which is due to image stitching. (M) Low contrast between gap region and cell tissue due to ripped tissue poses a challenge to cell detection. (N and O) Other factors. (N) Non-continuous cell borders. (O) Very bright staining artefacts.

Skeletal muscle and its adaptation to diverse stimuli plays a central role in various biological processes and disease states. Analysing the structural composition of skeletal muscle specimens is essential in many fields of research, ranging from basic developmental and physiological sciences to muscular and metabolic diseases like myopathies (Niemeijer et al., 2018). In preclinical models and studies in humans, one of the central elements in the characterisation of muscle specimens is the analysis of the muscle fibre size (fibre cross-sectional area (CSA)) (Niemeijer et al., 2018; Wurtzel et al., 2017; Salanova et al., 2014). CSA allows for the assessment of muscle hypertrophy, atrophy and weakness. Despite its importance, the quantification of such muscle cell characteristics is still often done manually by multiple blinded observers–the muscle fibres are delineated by hand using software such as ImageJ (Papadopulos et al., 2007). This is a time consuming and labour intensive task, especially if multiple cross sections have to be analysed for a research task. Methods which aid in automating the process are available, but require large amounts of manual error correction and are often not free, for example, the Zeiss AxioVison software. Automatic segmentation of muscle fibre cross-sections is a hard problem due to variation with regards to staining quality and noise. There are three major factors which pose challenges to automated muscle fibre detection approaches: brightness, borders, and image stitching. Firstly, in fluorescence-stained pictures, brightness of cell tissues varies strongly between and even within pictures (see Figs. 1A–1D). As an example, we observed a general trend of small fibres appearing brighter than big fibres. Biological reasons for the variation of brightness within a picture might be the autofluorescence of tissue, or the wheat germ agglutinin (WGA) used for staining in our images, which generally binds to glycoproteins of the cell membrane (Emde et al., 2014). It can thus also stain membrane vesicles, which would show up as small bright spots in the cytosol of the fibres. This form of noise within the fibres is differently distributed and is thus hard to filter out (see Figs. 1E–1G). Secondly, cell borders can contain weakly stained areas, which appear as holes, and in extreme cases the whole border may be hard to spot on the image (Figs. 1H–1K). Additionally, there are interstitial spaces (gaps) between the muscle fibre bundles in most cross sections, which are expected to be devoid of the staining protein. However, these areas often contain noise (Figs. 1L and 1M) and the resulting low intensity contrast between the gap and neighbouring cell tissue may hinder the correct detection of the cell borders. Another possible source of variation is the process of picture recording. When the whole image is reconstructed from multiple smaller pictures, these pictures are recorded separately using automatic brightness detection. The stitched cross section may thus contain sudden changes in intensity, where two small pictures recorded with different settings are assembled (Figs. 1L and 1M). All of these aforementioned factors are part of the challenge that needs to be overcome to realise effective and practical automatic fibre cross-sectional detection.

In the past years, methods for automatic or semi-automatic muscle cell detection on different staining techniques have been introduced. Smith & Barton (2014) (SMASH tool), Wen et al. (2018) (MyoVision tool), and Mayeuf-Louchart et al. (2018) (MuscleJ tool) created GUI-based methods which have the advantage of being freely available and usable. Miazaki et al. (2015) presented their method on immunohistochemical staining, and Mula et al. (2013) showed examples of both immunohistochemical staining and WGA fluorescence-staining. All aforementioned methods are purely computer vision (CV) based, that is, they combine several methods like watershed, morphological operations, or background correction.

Liu et al. (2013) presented a method on haematoxylin and eosin stained images which combines supervised learning (SL) with CV. To the best of our knowledge, there is no implementation of the suggested method available, and an implementation of the sophisticated methodology according to the description in the paper would, in our opinion, be a very challenging task to the average user. An error analysis regarding the deviation of the reconstructed areas from manual analysis is included, and the performance of the method seems very convincing. Similar to the CV-based methods above (with the exception of SMASH), the pipeline seems to be fit for batch processing of several images without user intervention, after parameters are tuned.

All of the above algorithms greatly facilitate cell detection in their respective settings, while they don’t seem to be applicable to images of different staining techniques. There is still no overall solution which addresses all three aforementioned challenges of automated muscle fibre detection available. Furthermore, the trade-off between automation and quick application on the one hand, and the adaptability and possibility of human intervention on the other hand is balanced differently between the methods, thus some of the methods might not fit the respective needs of a user.

The pipeline proposed in this paper is called the supervised learning-computer vision combined pipeline (SLCV), since it integrates SL and CV. It does require human intervention, but is very intuitively and quickly adjustable with respect to different staining methods and staining qualities, while being robust to noise and variation. The novelty of this work is firstly to combine the adaptability and robustness of machine learning methods with the accuracy of CV methods on fluorescence-stained images and secondly, to perform a comprehensive statistical error analysis with respect to cell separation and area reconstruction. We set up a comparison study between our SLCV pipeline and that of Mula et al. (2013), since it appears to be a very good purely CV-based automated fibre detection method. It states all methods in detail and arranges them in a three-step pipeline, which is very similar to the set-up of SLCV. Through the comparison of the respective steps’ outcome, we will be able to show the contribution of SL and CV to the segmentation quality, respectively, which is the third novelty of this work. We present both pipelines on the example of fluorescence-stained images, which Mula et al. have also used in their paper.

We begin by introducing both the SLCV pipeline and the pipeline of Mula et al., highlighting their key characteristics and similarities. Then, we describe how the test images were created, and define the error measurements of cell separation and area reconstruction used for the statistical analysis. Finally, we compare results we obtained using both pipelines on our image test set, and discuss these results.

## Methods

### SLCV pipeline

#### Step I: supervised learning

**Figure 2:**
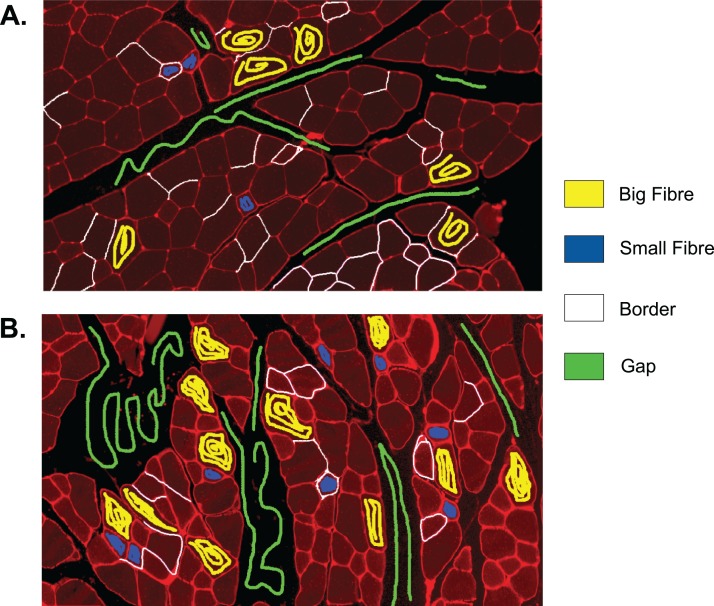
Training set of Ilastik classifier 1 out of 2. This classifier was applied to 25 out of 27 of the test set images. Yellow: class ‘big fibre’, blue: class ‘small fibre’, white: class ‘border’, green: class ‘gap’. (A) First training image. (B) Second training image.

The first step to identifying cells in the muscle cross-section picture is detecting the cell borders. To do this, a random forest SL model for pixel classification is used. Pixel classification models are available as one of several workflows in Ilastik–an open-source image analysis, classification, and segmentation software (Sommer et al., 2011). The trained model assigns every pixel of an image to one of several previously defined classes, which describe the different textures within the cross sections to be analysed. In fluorescence-stained images, we defined the classes to be ‘border’ (very bright and thin regions), ‘gap’ (big dark areas), ‘big fibre’ (bigger areas of low brightness), and ‘small fibre’ (smaller bright regions). Only pixels of the class ‘border’ are used for the following steps, but defining all four classes and training the model to distinguish them optimises the accuracy of the assignment of pixels to the ‘border’ class. The classification model is based on two selected image features, given in pixel (px) to describe the width of the image region used for their calculation: the eigenvalues of the Hessian of Gaussian with σ_texture_ = 1.6, 3.5, 5.0, 10.0 px which are used to detect regions of intensity changes, and Gaussian image smoothing with σ_intensity_ = 0.3, 0.7, 1.0, 1.6, 3.5 px. The pixel breadth values of σ are chosen from seven different values available in the feature selection dialogue of Ilastik, and are selected to cover a range of pixel breadths for each feature. Based on these features, the training is conducted on only few selected pixels from a set of example pictures. Ilastik features a visual interface, where the training pixels can be drawn on the image, making the model easily adjustable to different kinds of staining and quick and intuitive to create (see section ‘Recommendations for the Training of the Ilastik Classifier’ for how an Ilastik classifier is recommended to be created). All classification done in this work was performed using no more than small amounts of training on two images, as shown in Fig. 2. This Figure shows the training of classifier 1, which was applied to 25 test set images. The two training images in the Figure are examples of muscle fibre cross-sections, and were chosen such that they cover the common intensity and noise levels found in borders, gaps and fibres in the test set. The first image mostly contains noise-free gaps, fibres with low, equally distributed noise, and thin or faint borders. In contrast, the second image contains noisy gaps, very bright borders, and fibres with higher noise levels of different distribution. Classifier 2 was trained on two images in a similar fashion, and was applied to two test set images which showed much higher overall brightness and lower border quality than the other test images. Both images chosen for the training of classifier 2 also mainly contained high brightness fibres and thin and faint borders to match the test images and are shown in the section ‘Training set of Ilastik Classifier 2’.

**Figure 3:**
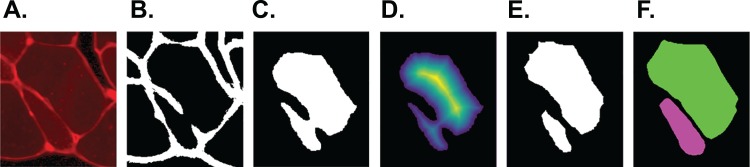
SLCV processing of an example image section. (A) Section of an original image after manual thresholding. (B) White pixels were classified as ‘border’ by the random forest segmentation model. (C) Initial cluster. (D) Distance transformation of the non-border pixels. The following thresholding was conducted with parameter τ = 0.3. (E) Results of applying the watershed algorithm to the thresholded image yields two final clusters. (F) GAC snake reconstruction of the two final clusters.

The output of step I are all pixels which Ilastik classified as ‘border’. We refer to these as the initial borders or initial clusters, with both representations being equivalent. We define the clusters as follows: clusters are the smallest objects (with respect to set inclusion) in the picture, which are each completely encapsulated by a continuous sequence of touching border pixels. The clusters represent initial fibre detection results and can consist of more than one true fibre if the borders contain holes. An example of the relation between borders and clusters is shown in Fig. 3, where Fig. 3B shows the borders resulting from the classification of Figs. 3A and 3C shows the cluster obtained from the borders in Fig. 3B.

#### Step II: watershed

The output of the previous step are the initial borders or clusters, where one cluster can contain one or multiple true fibres. The aim of this second step is to identify single fibres by refining or separating these initial clusters. This is achieved by filling holes in borders which were not completely detected by the SL pixel classification. First, very small holes are filled by dilating the borders. The binary image is then subjected to the following distance transformation: each non-border pixel within a cluster is given a value according to its minimal distance to a border pixel (Borgefors, 1986). This transformation yields a ‘hill-like’ structure, which has one local maximum if the cluster is round, or several local maxima, if the boundary is irregular and the cluster is thus likely to contain multiple single cells. A subsequent thresholding step removes small distance values and leaves connected components representing the maxima. Let *x* and *y* be the coordinates of a pixel inside an image *Î*. Then,

$$I(x,y): = \left\{ {\matrix{
{\hat I(x,y)} \hfill & {\quad {\rm{if}}\,\hat I(x,y) \ge {\rm{\tau }} \times {\rm{max}}(\hat I){\rm{,}}} \hfill \cr
0 \hfill & {\quad {\rm{else}}} \hfill \cr } } \right.$$

where *I* is the thresholded image *Î*. The bigger the threshold τ ∈ [0, 1] is selected, the more components result and the easier small irregularities within the cluster boundary lead to cluster separation. The connected components are subsequently input to the watershed algorithm (Chanussot & Lambert, 1999). The idea of the watershed algorithm is to ‘flood’, that is, to steadily extend all connected components outwards. Flooding is stopped in regions, where either the component touches a border pixel or two different components touch each other. A visualisation of input, distance transformation and output are shown in Figs. 3C–3E, respectively. The result of this step are the final cell clusters.

#### Step III: GAC snake

The previous two steps separated touching muscle cells in order to obtain single true cells. However, fibre area is lost in this process due to errors introduced by approximations in these steps and thus the aim of the last step is to accurately reconstruct the fibre area. To do this, the *geodesic active contours* (GAC) snake model–an evolving two-dimensional deformable curve–is used (Álvarez et al., 2010). It is based on a partial differential equation (PDE), which is solved repeatedly until its overall energy is minimised. The PDE has the form:

$${{\partial u} \over {\partial t}} = \underbrace {g(I){\kern 1pt} |\nabla u|{\kern 1pt} {\rm{div}}\left({{{\nabla u} \over {|\nabla u|}}} \right)}_{{\rm{smoothing\ force}}} + \overbrace {g(I){\kern 1pt} |\nabla u|{\kern 1pt} \nu }^{{\rm{balloon\ force}}} + \overbrace {\nabla g(I){\kern 1pt} \nabla u}^{{\rm{image\ attraction\ force}}}$$

It consists of three parts, which modify the deformable 2D curve *u*: a smoothing function, a balloon force and an image attraction term. The balloon force is controlled by the parameter ν and is used to expand the snake *u* outwards (or to contract it, if ν < 0), while the image attraction term *g*(*I*) draws the curve towards image features of interest and acts as a stopping criterion. We used the Marquez-Neila, Baumela & Alvarez (2014) implementation of the algorithm. One factor that distinguishes the GAC snake from other snake models is the usage of a morphological method for solving the PDE, which is quicker and numerically more stable than conventional numerical methods (Álvarez et al., 2010).

When edges in a picture are used as an image attraction force like in our case, then

(1) $$g(I) = {1 \over {\sqrt {1 + {\rm{\alpha }}|\nabla {G_{\rm{\sigma }}} \otimes I|} }},$$

which results in *g*(*I*) having its minima near regions with high intensity changes. In the above equation, ⊗ represents the convolution operator. There are two parameters to be set, and we chose α = 2,000 and σ = 2 after testing different parameters on the image set. Additional parameter choices are given in the section ‘Additional Parameter Choices in the Implementation’. The input of the snake algorithm is called *seed*, and its boundary represents the initial configuration of the curve *u*. Starting from the final clusters from step II as seeds, the cluster boundaries are expanded iteratively until they reach the cell borders (see Fig. 3F).

**Figure 4:**
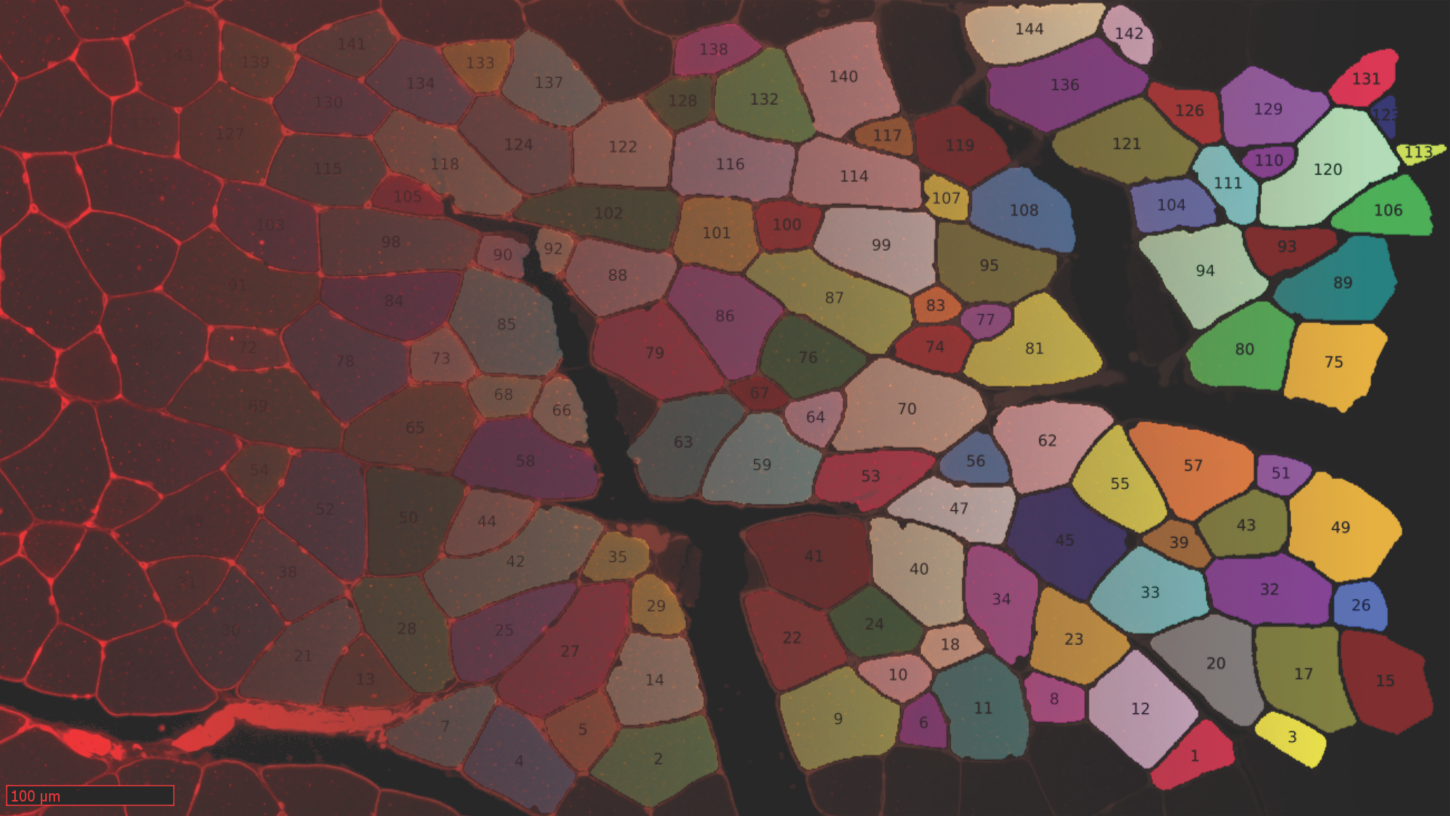
Example cross section after manual thresholding and the final processed image, blended into each other. Fibres touching the border are excluded from reconstruction.

In summary, the SLCV pipeline starts by applying the Ilastik random forest SL method to an input image in order to obtain the initial clusters. The subsequent steps are CV-based and correct and refine the SL output. The clusters are submitted to distance transformation and the watershed algorithm, with the aim of separating initial clusters into final single-cell clusters. The area which is lost in the separation process is then reconstructed by applying the GAC snake model to each final cluster, where the resulting fibres are obtained by growing the cluster using information from the original image. An example of an input and output of the pipeline is shown in Fig. 4. The output of SLCV contains the contours of all detected image fibres, which can be used to calculate the individual fibres’ characteristics such as CSA or feret diameter.

### Pipeline of Mula et al.

Similarly to the SLCV pipeline, Mula et al.’s (2013) pipeline consists of three phases which follow the same objectives as the SLCV phases, respectively. In the first step, the algorithm detects ridges, which are regions of intensity changes in the picture. This is achieved by first convoluting the input picture with a Gaussian kernel and then calculating the eigenvalues of the Hessian matrix of this convoluted image. From the eigenvalues, likelihood measures are obtained and subjected to automatic *Otsu Thresholding* (Otsu, 1979). In the second step, the initial borders are morphologically closed to fill very small holes. Then the resulting clusters are subjected to iterative erosion until their size falls below a given threshold. This strategy tends to split all but very round clusters into multiple regions. Thus, touching cells with medium-sized or big holes in their borders are separated, and a set of final clusters of similar size is obtained. The third step applies the snake algorithm to the final clusters to reconstruct the cell area. We implemented the pipeline of Mula et al. (2013) according to the description in the paper. Within the procedure, there were several parameters to be set. For some of them, a recommendation was given, in which case we set them accordingly. All other parameters were set such that we reached the best segmentation result on our test pictures. The parameters are given in the section ‘Additional Parameter Choices in the Implementation’. We also performed some changes on the pipeline: we changed the described multi-scale ridge detection of step I to single-scale detection with σ* = 0.7, because this parameter captured the borders best and minimised the noise in the resulting ridges. Furthermore, Mula et al. originally used the gradient vector flow (GVF) version of snake (Xu & Prince, 1997, 1998) in step III, which is similar to the GAC snake, because both use a smoothing term and an image attraction force. However, GVF snake lacks the balloon force term and does not always converge to the edges, if the initial seed is too small. Furthermore, if it is implemented according to the description in the original paper by Xu et al., it is less numerically stable than the GAC snake, since it doesn’t use morphological methods to solve the PDE. Therefore, to simplify the comparison of the two pipelines, we used the morphological GAC snake model instead of the GVF snake in our implementation of Mula et al.’s (2013) pipeline. As we have changed some details of Mula et al.’s description to optimise the performance on our image test set, we wish to highlight that the results of our implementation of the method are rather meant as a reference performance of the described high-quality CV-based strategy than as an evaluation of the strategy. The results of our implementation are necessary for estimating the contribution of SL and CV to the overall fibre detection quality.

### Picture test set

The animal sacrifice has been approved by Landsamt für Gesundheit und Soziales Berlin (G0303/15). Complete hindlimbs from male C57BL/6J mice aged 20 weeks were dissected and fixed in paraformaldehyde for 48 h at 4 °C to keep the knee joint and muscles in their natural position. The specimens were decalcified for 10 days in 14% *EDTA* at 4 °C on a shaker. After dehydration, joints were embedded in paraffin and serial cross-sections (five μm) through the whole hindlimb musculature were done. Cross sections were mounted on slides, stained with fluorescent-labelled WGA (Alexa-Fluor 555; Thermo, Waltham, MA, USA) and visualised using a slide scanner (Hamamatsu NanoZoomer; Hamamatsu, Hamamatsu City, Japan). The cross sections originate from different hindlimbs of several mice from multiple experiments, where some mice were healthy and some mice had previously undergone surgery on the knee.

The fluorescence-stained images created by this method have very similar properties to immunohistochemically stained images. Instead of fluorescence-labelled WGA, which binds to glycoproteins in the membrane, two types of antibodies are used to create immunohistochemically stained images. The first antibody binds to a specific protein which appears in the membranes of muscles. Commonly, laminin is targeted, but in some cases dystrophin is used as well. The second fluorescent protein binds to the first antibody to visualise the binding (Gao & McNally, 2015). Hence, noise and variation are comparable between both methods, and SLCV and Mula et al.’s pipeline can be compared using only one of the two types of staining.

Our test set contains 27 images of different staining quality, including noisy and low-quality images. This is evidenced by the examples from Fig. 1, which are all taken from the test set. The raw images are submitted to a manual thresholding step (see section ‘Pre/Postprocessing’). The contrast between gaps and fibres is maximised in this step in order to assure the best possible performance of the CV methods. In each image, the maximum threshold was chosen such that no holes appeared in any muscle fibre. The resulting images serve as the test set. Furthermore, a corresponding groundtruth picture was created for each test set image by an experienced biologist who manually delineated all fibres. Any fibre which was not completely contained in the picture was omitted in both the test set image and the groundtruth image in order to obtain an unbiased fibre sample.

### Error measure: cluster separation

A simple image gradient analysis method was applied to each image to obtain the border pixels. We then defined the reference clusters to be the smallest objects in the image fully encapsulated by border pixels, equivalent to the definition in the section ‘Step II: Watershed’. The reference clusters were grouped by the number of groundtruth cells *n* which they contain to represent separation difficulty. Only reference clusters with *n* > 1 were kept in the considered test set. Furthermore, cluster sizes for which there were fewer than five samples in the test set were omitted from the statistical analysis. We chose ridge detection with one additional dilation as the gradient analysis method (see section ‘Additional Parameter Choices in the Implementation’). To assess the separation quality of a particular pipeline, the reference clusters were compared to the fibres detected by the pipeline. The cluster separation error is a sensitivity measure, which is computed for each picture and each reference cluster size *n* using a contingency table as shown below:

**Table:** 

	Positive (P)	Negative (N)
True (T)	*a*	*c* = 2^*n*^ − 1 − *a* − *b* − *d*
False (F)	*b*	*d* = *n* − *a*

**Figure A1:**
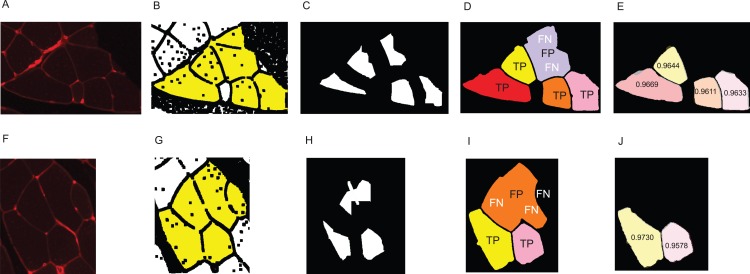
Visualisation of the error types. (A–E) Example for the cluster separation- and area reconstruction error of a reference cluster of size 6. (F–J) Example for both types of error of a reference cluster of size 5. (A, F) original image. (B, G) Yellow area shows the dilated reference cluster. (C, H) Seed obtained by automated reconstruction pipeline. (D, I) Reconstruction of the fibres. Visualisation of true positive, false positive, and false negative fibres as described in the section ‘Error Measure: Cluster Separation’. (E, J) DSC values of the correctly detected fibres. Images show an overlay of the groundtruth fibres and the reconstructed fibres.

The term *a* denotes the number of true positives, meaning the number of true cells in the reference cluster of size *n*, which were correctly detected by the algorithm. False negatives are denoted by *d* = *n*−*a* and describe the number of true cells not detected by the algorithm, either because the cell was missing completely in the result or because it could not be separated correctly. The false positives *b* are the number of clusters found by the algorithm, which contain more than one groundtruth cell. Finally, *c* is based on the size of the result space including every possible way to separate the reference cluster, from which all existing results are subtracted. An example of the cluster separation error is shown in the section ‘Visualisation of Both Error Types’, Fig. A1. The sensitivity is calculated as follows: ${\rm{sensitivity}}: = {a \over {a + d}}$. This measure captures how many cells within a reference cluster were separated correctly and is thus representative of the separation quality of a pipeline. In order to quantify the sensitivity difference between pipeline *pl*_1_ and pipeline *pl*_2_, we assume *H*_0_: ‘The average sensitivity of *pl*_2_ is ≥ the average sensitivity of *pl*_1_’. For each of the cluster sizes *n*, this hypothesis is tested by bootstrapping: 10^5^ bootstrap iterations are conducted on all reference cluster samples *N*. The *p*-value of this test is defined to be the number of bootstrap iterations in which *H*_0_ is true, divided by the total number of bootstrap resamples.

**Figure 5:**
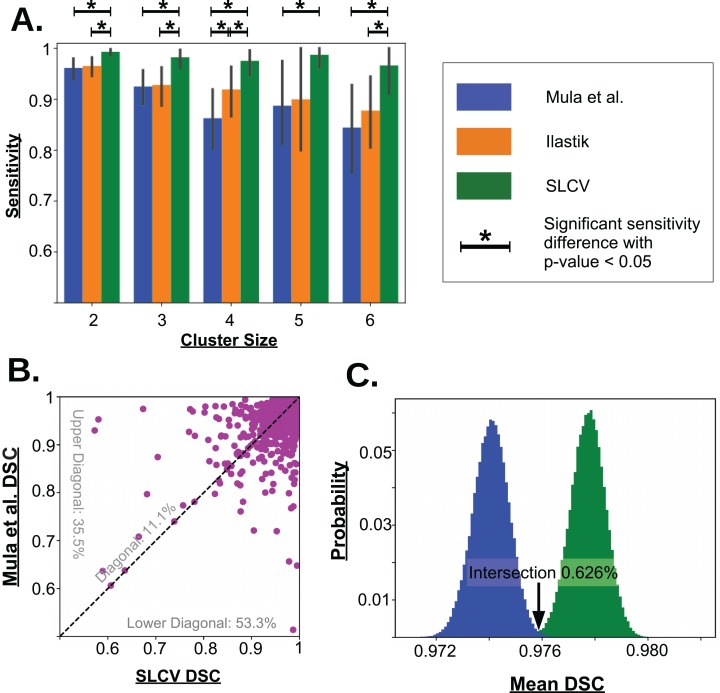
Visualisation of the cell separation and the area reconstruction error for all compared methods. (A) Average sensitivity values of all compared cell separation methods. * indicates a significant difference with *p*-value < 0.05. Error bars are determined by bootstrapping. Raw *p*-values for significant difference are given in Table A1 in section ‘Bootstrapping of Cluster Separation’, while raw sensitivity values are listed in Table 1. (B) DSC of Mula et al. and SLCV reconstructions of all correctly separated samples of the test picture set. (C) Mean DSC from 10^5^ bootstrapping iterations on the cell reconstruction results for Mula et al. and SLCV.

### Error measure: area reconstruction

The second type of error analysis in this work is based on the groundtruth of each test set image. The area of the groundtruth cell is compared to the calculated CSA for each cell that was correctly separated by all pipelines that are compared. This is done by calculating the Dice similarity coefficient (DSC), which is defined as follows:

(2) $${\rm{DSC}}: = {{2|X\bigcap Y|} \over {|X| + |Y|}},$$

where *X* is the area of the groundtruth cell and *Y* the area of the reconstructed cell (Dice, 1945). The DSC is a measure for the similarity of two areas and punishes deviations of a reconstructed cell from the original cell with respect to both size as well as location in the picture. An example of the area reconstruction error is shown in the section ‘Visualisation of Both Error Types’, Fig. A1. Differences in area reconstruction between two methods were quantified similar to sensitivity differences. We assumed *H*_0_: ‘The average DSC of Mula et al.’s (2013) pipeline is ≥ the average DSC of SLCV’, and tested it in each of the 10^5^ bootstrapping iterations. The *p*-value is the fraction of bootstrapping resamples in which *H*_0_ is true.

## Results

### Cluster separation

The aim is to observe, how well SL, CV, and combined SL and CV perform with regards to separation of clustered fibres (reference clusters) into their respective individual fibres. To understand the individual contribution, we chose an Ilastik classifier as a representative for SL, the Mula et al. pipeline as the representative of CV, and the SLCV method as the representative of the combined workflow. The quality of the cluster separation of each of these methods is given in Table 1.

**Table 1:** Sensitivity analysis: mean sensitivity values and 95% confidence intervals as visualised in Fig. 5A.

Cluster size	2 (*N* = 150)	3 (*N* = 58)	4 (*N* = 31)	5 (*N* = 8)	6 (*N* = 15)
Mula et al.					
Mean	0.92	0.85	0.73	0.78	0.69
95% CI	[0.88–0.96]	[0.78–0.92]	[0.60–0.83]	[0.60–0.95]	[0.49–0.88]
Ilastik					
Mean	0.93	0.86	0.84^$^	0.80	0.76
95% CI	[0.89–0.96]	[0.78–0.92]	[0.73–0.93]	[0.60–1.0]	[0.61–0.89]
SLCV					
Mean	0.99*^,^^†^	0.97*^,^^†^	0.95*^,^^†^	0.98*	0.93*^,^^†^
95% CI	[0.97–1.0]	[0.93–0.99]	[0.90–0.99]	[0.93–1.0]	[0.82–1.0]

**Notes:**

* Average sensitivity SLCV > Mula et al. with *p*-value < 0.05.

† Average sensitivity SLCV > Ilastik with *p*-value < 0.05.

$ Average sensitivity Ilastik > Mula et al. with *p*-value < 0.05.

**Figure A2:**
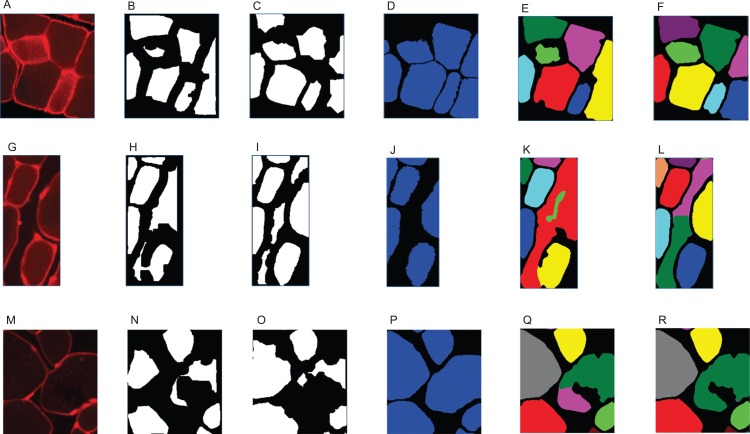
Examples of challenging cell reconstructions. (A, G, M) thresholded original picture, (B, H, N) final clusters produced by Mula et al.’s pipeline, (C, I, O) final clusters produced by SLCV pipeline, (D, J, P) groundtruth, (E, K, Q) Mula et al.’s segmentation result, (F, L, R) SLCV segmentation result. (A–F) Missing cell area in reconstruction of noisy cells due to small seeds, (G–L) merging of cells and gap regions due to low contrast, (M–R) oversegmentation of a fibre.

In the 27 test set pictures, 150 reference clusters of size 2, 58 clusters of size 3, 31 clusters of size 4, 8 clusters of size 5, and 15 cluster of size 6 were seen. Larger cluster sizes were also observed, however, they were excluded from the analysis as there were less than 5 occurrences in the test set pictures. Firstly, it can be seen that as the cluster size increases, there is a decrease in the average sensitivity, that is, larger clusters are harder to separate. For small cluster sizes, such as 2 and 3, Mula et al. (only CV) has an average sensitivity of higher than 85%. Interestingly, Ilastik (only SL) has a similar average sensitivity as Mula et al. Even though it appears that the sensitivity in Ilastik’s cluster separation decays slower than the sensitivity of Mula et al.’s pipeline with growing cluster size, there was no significant difference between the two methods (see Fig. 5A). Considering the SLCV method, it can be seen that the average sensitivity is significantly higher than in both Mula et al. and Ilastik. Only *n* = 5 is an outlier in this respect. Due to the low sample size of 8, the bootstrapping could not detect a significant difference between SLCV and Ilastik. Furthermore, the average sensitivity of the SLCV pipeline only decreased by approximately 0.05 between cluster sizes two and six. In comparison, Mula et al. and Ilastik decreased by approximately 0.2 over the same range. This shows that the combination of both SL and CV is significantly better at cluster separation than either SL or CV alone, and that SLCV has a high chance of accurately separating even large fibre clusters. In contrast to incomplete cluster separation, we also observed oversegmentation in both pipelines causing separation errors (see Figs. A2M–A2R). That is, a cluster representing a true cell is sometimes erroneously separated into two or more clusters. In the watershed algorithm, oversegmentation is a known problem (Li & Xiao, 2004; Gauch, 1999; Liu et al., 2013), while in Mula et al.’s pipeline, the cause are errors introduced by erosion.

### Areas

Now, the aim is to study if combining SL and CV results in a more accurate reconstruction of muscle fibre area than using CV only. In this analysis step, only fibres which were correctly separated by all compared methods were considered in order to clearly separate the cell separation error from the area reconstruction error. However, it has to be noted that omitting non-separated cells from the analysis underestimates the consequences that incorrect cell separation has on area reconstruction, that is, the combined effect of both errors. To address this phenomenon, an additional analysis step is presented in the section ‘Analysis of the Combined Cluster Separation-Area Reconstruction Error’. In this section, we only compare the Mula et al. reconstruction with the SLCV reconstruction. The Ilastik method which we included in the cluster separation analysis is omitted, since Ilastik yields less correctly separated single fibres than SLCV, but these fibres share the same cluster shape and size as in SLCV and thus, both methods are equivalent in this comparison.

There were a total of 2,603 fibres correctly separated by both Mula et al. and SLCV. For each fibre, the DSC (section ‘Error Measure: Area Reconstruction’) between the groundtruth and the GAC snake-reconstructed fibre were calculated. The results are shown in Fig. 5B. It was found that approximately 11.1% of the fibres had a similar DSC in both the Mula et al. and the SLCV pipeline. Furthermore, approximately 35.5% of fibres had a higher DSC if they were reconstructed by the Mula et al. pipeline. The remaining fibres (approximately 53.3%) had a higher DSC in the SLCV pipeline results.

Regarding the average reconstruction quality of fibres, the average DSC over the 2,603 fibres was approximately 0.9741 for Mula et al. and 0.9778 for SLCV (see Table 2). The two averages are very close, however, when the samples are bootstrapped, it becomes clear that the average DSC of SLCV reconstructions is significantly better (see Fig. 5C; Table 2). The bootstrapping procedure draws–with replacement–a new set of fibres from the original set in each iteration and calculates the average DSC every time. A small *p*-value obtained by this procedure (as explained in the section ‘Error Measure: Area Reconstruction’) confirms that the difference between two methods is not due to chance. Hence, combining SL and CV produces a better reconstruction of a muscle fibre than a purely CV-based method. Considering that Mula et al. and the SLCV method both used the same GAC snake implementation, the gain observed in the reconstruction quality must be attributed to the size and shape of the cluster produced in the cluster separation step of the respective method.

**Table 2:** Area reconstruction analysis: comparison of the area reconstruction quality of SLCV and Mula et al. Including only the reconstruction results of cells that were separated correctly by both pipelines.

	Mula et al.	SLCV
Average DSC	0.9741	0.9778*
95 % CI	[0.9727–0.9754]	[0.9764–0.9790]
Original sample size	2,628	2,735
Adapted sample size	2,603	2,603

**Note:**

* Average DSC SLCV > Mula et al. with *p*-value < 0.05.

## Discussion

In this work, we introduced a semi-automatic muscle cell segmentation pipeline, which is robust against variation of image features. The usage of the random forest classifier as a SL technique is critical, as it can cope with imaging variation and noise much better than CV strategies, which solely use intensity changes for border detection. A beneficial effect of this is that SLCV does not require any preprocessing of the raw images, which a CV-only pipeline as Mula et al. does, since otherwise fibres positioned on the outside of muscle fibre bundles can be lost due to insufficient contrast. An example is shown in the section ‘Examples for Challenges in Muscle Fibre Detection’, Figs. A2G–A2L. However, preprocessing using an intensity threshold is still recommended to prevent or reduce the issue of gaps being detected as muscle fibres.

We experimented with other SL methods that could be used in the first phase to replace the random forest model. We trained a convolutional neural network (CNN) on multiple training images and found that no additional gains in the quality of the borders could be observed (section ‘Alternatives for Step I of the SLCV Pipeline’). The pipeline is thus adaptable to different SL methods, but the random forest performs best among the methods tested.

The SLCV pipeline is quickly and intuitively adaptable due to the training and parameter tuning process: Training requires no more than drawing a few lines onto a training image. Tuning of the pipeline is very user-friendly, because apart from the training process, there is only one parameter which needs to be set in order to obtain a good segmentation quality, which is the distance transformation cut-off within the watershed algorithm. In contrast, Mula et al.’s pipeline needs several parameters to be tuned in the first two phases. The GAC snake algorithm used for both pipelines contains additional parameters, which were relatively easy to set for our test images, since the best setting with respect to the image test set was close to the parameters given in the implementation of Marquez-Neila, Baumela & Alvarez (2014). This makes the SLCV pipeline not only adaptable to different data sets, but also to different staining methods: The classes of the segmentation classifier can be changed to represent the different typical textures in images created by the staining method. Furthermore, the usage of SLCV is not restricted to muscle segmentation problems, but can be applied to other problems, for example heart- or nerve cell segmentation or the detection of any other tubular or rounded structure in stained images.

A positive characteristic is that the cell separation quality of the pipeline can be as accurate as the user wishes. That is, little training on few pictures is sufficient to obtain a good result, but if a perfect separation is required, the pipeline can be tuned to achieve this result by more training or by splitting the picture set into multiple subsets of similar pictures, with one classifier for each subset. This splitting could be automised by characteristics like average picture intensity or intensity distribution, but it can also be done manually.

One limitation is that it is not possible to use the pipeline without human input. However, once the respective classifiers have been created for the user’s different classes of imaging data and an appropriate parameter for watershed is chosen, the algorithm can automatically be applied to new data. Another limitation are the artefacts that are introduced because of the oversegmentation of the watershed algorithm. In Mula et al.’s pipeline, where the oversegmentation originates from erosion, these false segmentation results can not be removed easily due to the high level of automation of the pipeline. An example is shown in the section ‘Examples for Challenges in Muscle Fibre Detection’, Figs. A2M–A2R. In SLCV, oversegmentation can be lowered or even completely avoided by adding more training lines to Ilastik or by splitting the image set as described above. However, a way to improve the algorithm in the future would be to find a strategy that can circumvent the oversegmentation problem which is inherent to the watershed algorithm (cf. Bieniecki, 2004; Gonzalez, Meschino & Ballarin, 2013; Acharjya & Ghoshal, 2013), and which is suitable for the type of images used in the SLCV pipeline.

We found that the size and shape of the cluster input to the snake algorithm has an impact on the area reconstruction quality. In the fluorescence-stained images processed here, as well as in the immunohistochemically stained images with similar characteristics, this is very likely due to the noise present in the fibres. This noise can disturb the image attraction force used in the GAC snake algorithm, such that the area can not expand to the true cell borders. Since the fibre separation strategy used in the SLCV pipeline does not involve shrinking of the cell-cluster, the resulting final clusters are bigger than in shrinkage-based separation methods such as erosion (step II in Mula et al.’s pipeline). These bigger clusters are already close to the original area and thus yield a better DSC, if the cell can not be reconstructed correctly. Another factor which contributes to the bigger size of final clusters in the SLCV pipeline is that the SL step without further CV correction already provides reasonably good cluster separation. Thus, many clusters already represent single fibres and are unchanged after the watershed algorithm. An example is shown in the section ‘Examples for Challenges in Muscle Fibre Detection’, Figs. A2A–A2F.

In the section ‘Areas’, we showed that due to the favourable characteristics of the seeds created by SL combined with CV on fluorescence-stained images, the area reconstruction of SLCV is significantly better when compared to pure CV. However, since the actual difference between the DSC values is very small, the first type of error—the cluster separation error—is much more important in practice to minimise the bias introduced to the reconstructed muscle CSA.

## Conclusion

In this paper, we have introduced a semi-automatic muscle cell segmentation pipeline, which is robust against variation of image features. Due to the usage of the Ilastik SL classifier, the pipeline is quickly and intuitively adjustable to different staining qualities and furthermore, it is applicable to different staining techniques and can be adapted to segment cells in various tissues.

We have compared SLCV to the CV-based segmentation pipeline of Mula et al. (2013) to quantify the contribution of both SL and CV to the quality of the final reconstruction result. With regards to cell separation quality, we showed that SL (step I of the SLCV pipeline) performs as well as two CV methods combined (step I and II of two Mula et al.’s pipeline) and thus outperforms pure CV. However, when SL was combined with a CV correction step (the second step of SLCV), a significant improvement in the separation quality could be seen with regards to both CV and SL. Hence, learning alone is a powerful method, but to reach optimal performance, it has to be combined with CV.

With regards to area reconstruction, the combination of SL and CV also leads to a significant improvement compared to CV methods only, which is due to the more favourable characteristics of the final clusters created by SLCV on fluorescence-stained images, and leads to an improved robustness to noise. Thus, combining SL and CV creates a significant improvement with respect to all muscle fibre detection quality criteria used in this work and is thus a superior method to both purely SL- and CV-based methods.

## Appendix

### Recommendations for the training of the Ilastik classifier

We recommend the training of a segmentation classifier on two cross-sectional pictures, which taken together contain the lower and upper end of the range of border intensity, cell plasma intensity, cell plasma structure, noise amount, and noise distribution of the pictures that the classifier will be applied to. However, if the range of intensities is very big, the classifier will lose accuracy and two classifiers should be trained on the lower and upper part of the intensity range, respectively. Training pixels for ‘border’ should be drawn in with a relatively small brush, and a border should be drawn in one continuous stroke. It is especially recommended to add borders which contain areas of visibly lower staining intensity to the training pixels, see Figs. 1H–1K. Additionally, borders which are not continuous due to torn tissue or staining artefacts like in Figs. 1N and 1O should imperatively be added to the training set, drawn as a continuous object. In other words, borders which are flawed in the raw picture should be drawn in the way they would optimally look like. The training dialogue features a live update, which can be used to check if holes remain in the training picture borders. If so, these borders should be added to the training set. Big fibre and small fibre training should include cells with different structure (different intensity and noise distribution). The whole cell is recommended to be covered with training pixels, and border pixels have to be excluded. The live update should be used to check if border pixels are found within cell or gap regions. If so, it is recommended to add a few more cell training pixels, drawing one fibre at a time. Training on gap regions can be sparse, but should include intensity changes and artefacts that may be found in the gap regions, such as very bright objects or background noise. In general, artefacts should be labelled as the object that they are supposed to represent. When the classifier is applied to the training images and the result contains little to no holes in the borders, and no border is predicted in gap or fibre regions, the classifier is fit to be applied to test set images. If this state is not reached or can only be reached by drawing a lot of training lines, the training images might be too different from each other and at least one image should be replaced.

### Training set of Ilastik classifier 2

**Figure A3:**
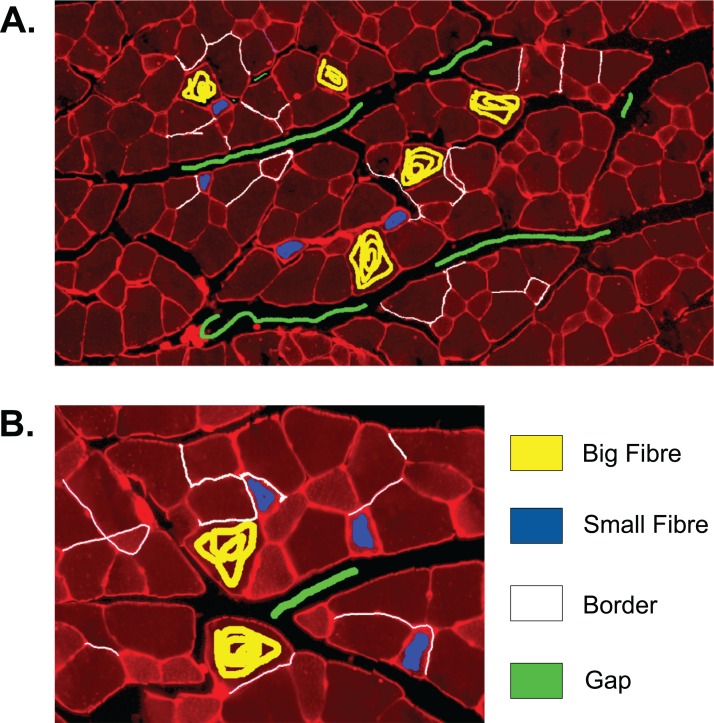
Training set of Ilastik classifier 2 out of 2. This classifier was applied to 2 out of 27 of the test set images which were of higher brightness and contained lower quality borders than the other test set images. Yellow: class ‘big fibre’, blue: class ‘small fibre’, white: class ‘border’, green: class ‘gap’. (A) First training image. (B) Second training image, which is a section from a full image.

The full training set for classifier 2 is given in Fig. A3. Figure A3B shows a section that was cut out from a cross-sectional image, since no training lines were added to the rest of the image. The size of the section remains the same as in the original image.

### Additional parameter choices in the implementation

#### Ridge detection (Step I in Mula et al.)

In contrast to the description in Mula et al., we performed single-scale ridge detection, where the scale parameter is denoted by σ*. We chose σ* = 0.7. We first constructed the Hessian matrix *H*:

(3) $$H(x,y,{{\rm{\sigma }}^*}) = \left[ {\matrix{
{{{{\partial ^2}{I_{{{\rm{\sigma }}^*}}}(x,y)} \over {\partial {x^2}}}} & {{{{\partial ^2}{I_{{{\rm{\sigma }}^*}}}(x,y)} \over {\partial x\partial y}}} \cr
{{{{\partial ^2}{I_{{{\rm{\sigma }}^*}}}(x,y)} \over {\partial x\partial y}}} & {{{{\partial ^2}{I_{{{\rm{\sigma }}^*}}}(x,y)} \over {\partial {y^2}}}} \cr
} } \right],$$

where *I*_σ*_(*x*, *y*) = *I*(*x*, *y*) ⊗ *G*(*x*, *y*, σ*) and ⊗ represents the convolution operator. *G*(*x*, *y*, σ*) is the two-dimensional Gaussian kernel:

(4) $$G(x,y,{{\rm{\sigma }}^*}) = {1 \over {\sqrt {2{\rm{\pi }}} ({{\rm{\sigma }}^*})}} {e^{-{{{x^2} + {y^2}} \over {2({{\rm{\sigma }}^*}{)^2}}}}}$$

Then, the two eigenvalues λ_1_, λ_2_ of *H* with |λ_1_| > |λ_2_| were computed and each ridge obtained a likelihood measure:

$${r_{{{\rm{\sigma }}^*}}} = \left\{ {\matrix{0 \hfill & {\quad \text{if}\ {{\rm{\lambda }}_1} > 0} \hfill \cr{{e^{ - {{{R_B}} \over {{{\rm{\alpha }}^2}}}}}\left( {1 - {e^{{{ - {S^2}} \over {{{\rm{\beta }}^2}}}}}} \right)} \hfill & {\quad \text{else}} \hfill \cr} } \right.$$

where ${R_B} = \left| {{{{{\rm{\lambda }}_2}} \over {{{\rm{\lambda }}_1}}}} \right|$, *S* = λ_1_^2^ + λ_2_^2^, and we chose α = 0.5 following a recommendation from Frangi et al. (1998) and β = 0.03.

#### Erosion (Step II in Mula et al.)

Ridges were submitted to a closing operation, where we chose the kernel to be of rectangular shape with size 11 × 11 px. After that, Mula et al. state that the inverse of the resulting edge map was used to detect the clusters. We implemented this as a dilation step carried out in two iterations with a rectangular 3 × 3 px kernel followed by the inversion of the edge map and a contour detection algorithm. Each detected cluster was then iteratively eroded with an elliptical kernel as specified in Mula et al., of size 8 × 8 px, until it was smaller than a certain threshold. We used the exemplary threshold of 5,000 px given in Mula et al. Additionally to the procedure described in the paper, we filtered out clusters which were smaller than 500 px to remove artefacts.

#### GAC snake (Step III in SLCV and Mula et al.)

Additional parameters for the snake algorithm are ν, μ, and θ. ν determines the strength of the balloon term and is used as ν = −1 for a deflating balloon and as ν = 1 for an inflating balloon in the implementation of the GAC snake by Marquez-Neila, Baumela & Alvarez (2014). We used ν = 1. μ controls the number of repetitions of the smoothing step in every iteration of snake. We chose μ = 3 to obtain a smooth curve. θ is related to the discretization of *g*(*I*) and used to control the smoothing operation strength at different points of the curve. We set θ = 0.3 analogous to the parameter setting in all test cases given in the implementation by Marquez-Neila et al. For the algorithm to work correctly, the final cluster has to be completely enclosed within the boundaries of the original cell, since we chose the balloon force to have an inflating effect. Furthermore, the bigger the cluster is, the more robust the area detection is for muscle cells which contain high concentrations of the staining protein. As mentioned in the Discussion, this form of noise, especially close to the muscle bundle border, disturbs the image attraction term and leads to an early stop of the snake contour and thus to an incomplete area reconstruction (see Figs. A2A–A2F).

### Visualisation of both error types

Figure A1 shows some examples of the cluster separation error and the area reconstruction error. Figures A1B and A1G show the reference cluster obtained by applying a simple image gradient analysis method, consisting of *n* = 6 and *n* = 5 true fibres, respectively. After reconstruction of the fibres, the sensitivity is calculated using the number of true positive fibres as described in the section ‘Error Measure: Cluster Separation’. The sensitivity in Fig. A1D is 0.67 and 0.4 in Fig. A1I. After determining the cluster separation error, the area reconstruction error is calculated for every true positive fibre by overlaying it with the groundtruth fibre, that is, the DSC is determined as described in the section ‘Error Measure: Area Reconstruction’.

### Pre/Postprocessing

Muscle fibre bundles inside a picture are usually separated from each other by an interstitial area which does not contain stained cells and which we refer to as gap region. In many pictures, this area contains a certain concentration of the stained protein. The more protein the region contains, the brighter it appears in the picture and the lower the contrast to the staining intensity of muscle cells. Consequently, it is harder to properly detect the fibre bundle boundaries. While the training step in our SL strategy can be adapted to correct for the latter phenomenon, it is problematic for CV-based pipelines such as that of Mula et al. and often results in gaps in the muscle fibre bundle boundary, such that outer muscle cells of the bundle may not be identified correctly (see Fig. A2G–A2L). Furthermore, staining noise in gap regions makes it impossible to automatically distinguish gaps from muscle cells, such that spurious muscle cells detected in gap regions would have to be removed in a post-processing step. Hence, a suggested strategy is to manually choose an intensity threshold τ ∈ [0, 255] (for 8-bit pictures) with

$$I(x,y): = \left\{ {\matrix{{\hat I(x,y)} \hfill & {\quad {\text{if}}\ \hat I(x,y) \ge {\rm{\tau }}} \hfill \cr0 \hfill & {\quad \text{else}}\ \hfill \cr} } \right.$$

and to exclude regions of value zero from the cell detection. We have also created a preliminary strategy for automatic thresholding to further reduce the necessary amount of manual work, which uses the histogram of image intensity values. The algorithm attempts to detect the peak representing gap intensity values in order to use it as a threshold value. However, the appearance of the histogram mainly depends on the bin size and automatic thresholding is a hard problem in general.

### Bootstrapping of cluster separation

Table A1 contains the *p*-values of sensitivity differences between the Ilastik random forest model, the SLCV pipeline and Mula et al.’s pipeline (shown in Fig. 5A in the section ‘Cluster Separation’). These *p*-values were obtained by bootstrapping, as described in the section ‘Error Measure: Cluster Separation’. A *p*-value smaller than 0.05 means that the sensitivity of *pl*_1_ (left term) is significantly higher than the sensitivity of *pl*_2_ (right term).

**Table A1:** Raw *p*-values of the cell separation sensitivity differences in Fig. 5A, in the section ‘Cluster Separation’.

*n*	*p*-value SLCV vs. Mula	*p*-value SLCV vs. Ilastik	*p*-value Ilastik vs. Mula
2	1.5 × 10^−4^	7 × 10^−5^	0.383
3	5 × 10^−5^	7 × 10^−5^	0.466
4	≪ 0.05	9.2 × 10^−4^	0.0152
5	0.021	0.058	0.462
6	≪ 0.05	8 × 10^−5^	0.275

**Note:**

The *p*-values are obtained by bootstrapping, *H*_0_: ‘The average sensitivity of pipeline *pl*_2_ (right term) is ≥ the average sensitivity of pipeline *pl*_1_ (left term)’.

**Figure A4:**
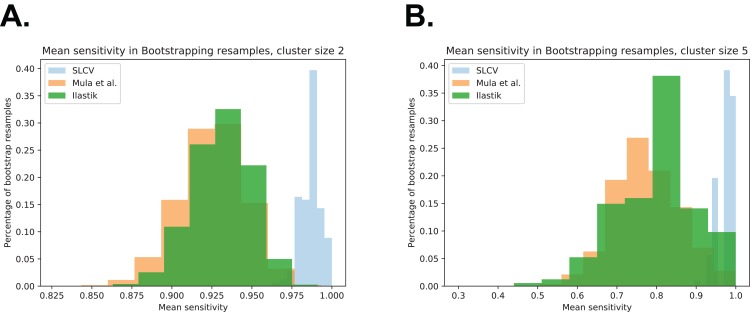
Bootstrap distributions of average sensitivity values for different cluster sizes. (A) Cluster size 2. (B) Cluster size 5.

Figure A4 shows the bootstrapping distribution over 10^5^ resamples with respect to cluster separation (similar to Fig. 5C in the section ‘Areas’, which show the same distributions with respect to DSC). The Figures contain the bootstrapping results of the three pipelines with respect to cluster separation, and they are included to visualise the small *p*-values given in Table A1 above.

### Analysis of the combined cluster separation-area reconstruction error

The Kullback–Leibler (KL) divergence is a measure for the distance between two probability distributions (Kullback & Leibler, 1951). If the distributions *P*(*x*) and *Q*(*x*) are discrete and they are both defined on the same space, the KL divergence is defined as follows (MacKay, 2005):

(5) $${D_{{\rm{KL}}}}(P||Q) = \sum\limits_x P(x)\ {\rm{ log}}{{P(x)} \over {Q(x)}}.$$

The distribution of groundtruth cell areas *P*(*x*) was compared to the distributions of areas *Q*(*x*) reconstructed by SLCV and Mula et al.’s pipeline, respectively. The histograms of the distributions were calculated using interval bin sizes *h_b_* = {50, 100, 200, 500, 1 × 10^3^, 2 × 10^3^, 5 × 10^3^, 1 × 10^4^, 2 × 10^4^}.

**Figure A5:**
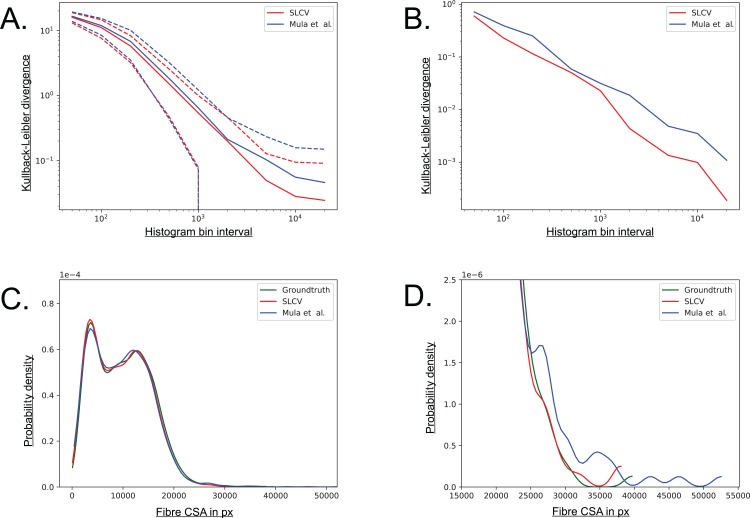
Visualisation of the combined cell separation-area reconstruction error. (A) Kullback–Leibler divergence of SLCV and Mula et al. reconstructed cell area distributions from groundtruth cell area distribution. Solid line represents the mean KL divergence over all images, dotted lines represent the standard deviation. Both axes are plotted logarithmically. (B) Kullback–Leibler divergence of SLCV and Mula et al. reconstructed cell area distributions from groundtruth cell area distribution of all images. (C) Kernel density estimation of cell areas over all images. (D) Zoomed in tail region from C.

Figure A5A shows the mean KL divergence and the standard deviation calculated over all test set images. The KL divergence shrinks exponentially with growing bin interval size, but the SLCV reconstruction shows a consistently smaller deviation from the groundtruth cell area than Mula et al.’s reconstruction, while the standard deviations are similar. The difference between the SLCV deviation- and the Mula et al. deviation from the groundtruth grows with growing bin interval size. Figure A5B shows the same calculation, only for the cell area distribution of all images taken together. The KL divergence is smaller than in Figure A5A, where the mean value over the single images was considered. However, the shrinking behaviour with growing bin interval size is similar in both Figures, while the difference between the curves fluctuates more in Fig. A5B. In conclusion, SLCV consistently reconstructs cell areas better than Mula et al. for all histogram bin intervals considered and in both single images and the overall test set; as it is always closer to the groundtruth cell area distribution.

Figures A5C and A5D show a kernel density estimation of the cell area distributions over all test set images. A kernel of bandwidth *bw_k_* = 0.2 was used to estimate the densities of the histograms, which were constructed with bin interval *h_b_* = 800. As seen in Fig. A5C, both SLCV and Mula et al. are close to the original cell area distribution, while SLCV shows less deviation from the original distribution in the peaks. In Fig. A5D, the tail of the distributions is zoomed in. Again, SLCV is closer to the groundtruth, while Mula et al. shows a longer and more pronounced tail, resulting from the bigger cell separation error.

If individual test set images are considered, the deviation from the groundtruth distribution of SLCV and Mula et al. varies more (data not shown): Mula et al.’s (2013) distribution shows a shift to the left in a few images, which represents a tendency to incomplete area reconstruction in these images. In most cross sections, both reconstructed area distributions have a tail on the right side, while the tail of Mula et al.’s curve is more pronounced. Overall, SLCV is mostly close to the original distribution, while Mula et al.’s area distribution tends to deviate from the original shape more due to the longer tail.

In summary, a higher cell separation error leads to a bigger area reconstruction error, hence a low cell separation error is a necessary condition for an automated cell separation pipeline to create cell reconstructions of high quality and to yield good CSA estimates.

### Examples for challenges in muscle fibre detection

Two representative examples for challenges we observed in automated muscle fibre detection are shown in Fig. A2. Row A shows incompletely reconstructed cells, which appear in cases where the image attraction force used in the GAC snake algorithm is disturbed by large amounts of noise. The final clusters created by the SLCV pipeline are bigger and are thus not affected as much as the seeds created by Mula et al.’s pipeline. Row B shows an example of the effect that a noisy gap region can have on cell separation. The border between fibre and gap is not bright enough to be fully detected, such that the cell is eventually lost in the purely CV-based pipeline. The SL step of SLCV was able to classify the border, because its training included noisy gap regions. In most images, this phenomenon can be avoided by preprocessing with a thresholding step. Row C shows an example of the oversegmentation of both Mula et al.’s (2013) erosion step and the watershed algorithm. In this case the problem arises because there is a tear in the tissue of one of the cells.

### Alternatives for Step I of the SLCV pipeline

**Figure A6:**
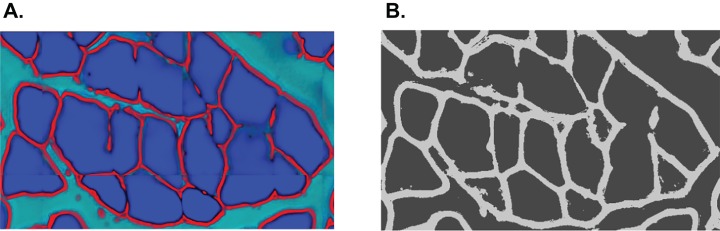
Comparison of supervised learning methods to create the initial borders. (A) Output of the CNN. Red: borders, green: gap, blue: muscle fibre. (B) Output of Ilastik. Only class ‘border’ is shown in light grey.

Alternative SL techniques can also be used for the detection of the initial borders. We trained a CNN on multiple images and could not detect any quality gains in the detected borders. An example of the output of the CNN and that of the Ilastik random forest classifier can be seen in Fig. A6.

## Additional Information and Declarations

### Data Availability

The following information was supplied regarding data availability: Raw images, the SLCV code and images processed using the SLCV pipeline and Mula et al.’s pipeline are available on GitHub: https://github.com/AnikaNovikov/SLCV.
